# The Intestinal Epithelium – Fluid Fate and Rigid Structure From Crypt Bottom to Villus Tip

**DOI:** 10.3389/fcell.2021.661931

**Published:** 2021-05-20

**Authors:** Vangelis Bonis, Carla Rossell, Helmuth Gehart

**Affiliations:** Institute of Molecular Health Sciences, ETH Zurich, Zurich, Switzerland

**Keywords:** intestine, stem cell, plasticity, differentiation, single cell, organoid, regeneration, cancer

## Abstract

The single-layered, simple epithelium of the gastro-intestinal tract controls nutrient uptake, coordinates our metabolism and shields us from pathogens. Despite its seemingly simple architecture, the intestinal lining consists of highly distinct cell populations that are continuously renewed by the same stem cell population. The need to maintain balanced diversity of cell types in an unceasingly regenerating tissue demands intricate mechanisms of spatial or temporal cell fate control. Recent advances in single-cell sequencing, spatio-temporal profiling and organoid technology have shed new light on the intricate micro-structure of the intestinal epithelium and on the mechanisms that maintain it. This led to the discovery of unexpected plasticity, zonation along the crypt-villus axis and new mechanism of self-organization. However, not only the epithelium, but also the underlying mesenchyme is distinctly structured. Several new studies have explored the intestinal stroma with single cell resolution and unveiled important interactions with the epithelium that are crucial for intestinal function and regeneration. In this review, we will discuss these recent findings and highlight the technologies that lead to their discovery. We will examine strengths and limitations of each approach and consider the wider impact of these results on our understanding of the intestine in health and disease.

## Introduction

As a single-layered columnar epithelium, the cell lining that covers the digestive tract appears deceptively simple. However, the epithelium and the underlying mesenchyme is exquisitely structured. This is necessary to protect stem cells from the harsh environment inside the intestinal lumen, to optimize nutrient uptake and to maintain a seamless barrier that protects against mechanical stress, low pH and pathogen invasion. These diverse requirements led to the evolution of crypt and villus domains, which support regeneration and nutrient uptake respectively. Within each domain, we find even more refined zonation with certain cell types and functions appearing only in specific positions along this crypt-villus axis. The existence of refined spatial organization is unexpected, when we consider the other defining characteristic of the epithelium: continuous proliferation. The epithelium turns over every 2–4 days in mice and every 2–5 days in humans (Darwich et al., 2014). The same continuously dividing stem cell population at the bottom of intestinal crypts generates all intestinal epithelial cells. Their offspring moves from the crypt up the villus to be eventually shed into the lumen at the villus tip. Therefore, the intestinal epithelial cell sheet is in continuous motion and moves up to half a millimeter per day. Despite this movement, the general spatial organization in the epithelium remains static. This is only possible since positional cues repeatedly induce and suppress cell fates in individual cells along their journey toward the villus tip. As a result, cells of the intestinal epithelium have to remain plastic and highly responsive to environmental cues that instruct their fate and function throughout their lifetime. In this review, we will explore this intricate link between spatial organization and plasticity in health and disease. We will highlight recent findings and discuss the advantages and disadvantages of the new methods and technologies that uncovered the full extent of structured diversity in the intestine.

### Form Follows Function

**FIGURE 1:**
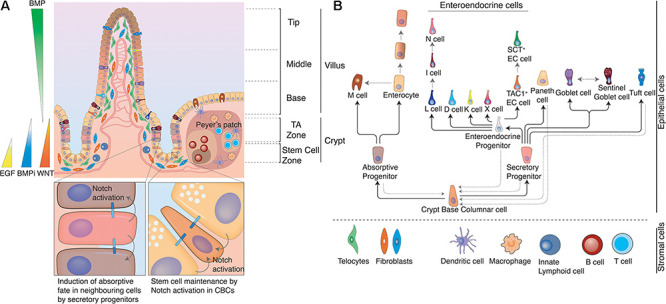
Structure of the intestine. **(A)** The intestine is organized in crypt-villus units. At the bottom of the crypt, in the stem cell zone crypt-base columnar cells (CBCs) act as stem cells of the tissue and are intercalated between Paneth cells. Paneth cells are the primary niche of CBCs and provide them with Notch ligands, EGF, and WNTs to support their continuous proliferation. At the same time Paneth cells also produce anti-microbial products to protect CBCs. In the Transit Amplifying zone (TA zone) the highly proliferative absorptive and slow dividing secretory progenitors differentiate to their respective lineage. The ratio between absorptive and secretory progenitors is controlled *via* lateral inhibition. Epithelial cells moving from the crypt bottom toward the villus encounter several opposing signaling gradients, among them WNT and BMP. WNT signals, which are necessary for the stemness of CBCs, are higher at the crypt bottom and gradually decrease toward the villus, while increasing BMP levels induce differentiation and gradual fate changes as cells rise up toward the villus tips. These signaling gradients are shaped by mesenchymal populations, such as fibroblasts or telocytes. Distinct populations with differing secretory profiles constitute the mesenchymal stem cell niche adjacent to crypts or induce continuous fate changes along the villus. Gray solid arrows indicate cells with Notch activity. **(B)** Cell fate determination in the intestinal epithelium. Once CBCs leave the stem cell zone, they start to differentiate either toward the absorptive or the secretory fate depending on Notch signals. Secretory progenitor cells can give rise to Paneth cells, goblet cells, Tuft cells and enteroendocrine cells, while absorptive progenitors can give rise to microfold cells and enterocytes. However, fate changes are not unidirectional and can be reverted upon appropriate environmental stimuli, such as tissue damage. Likewise, certain intestinal epithelial populations (e.g., enterocytes, EE cells, and goblet cells) dynamically acquire and lose different functions and thus cell identities in the course of their lives due to the instructive capacity of changing environments that they traverse as they move along crypt and villus. Black solid arrows indicate cell fate decisions during the differentiation process and gray dotted arrows indicate documented plasticity events by distinct cell populations.

Evolution integrated the conflicting needs for maximized absorption and barrier function by creating crypts and villi (see Figure 1A). Villi are capillary-rich protrusions into the intestinal lumen of 1.6 mm (proximal) to 0.5 mm (distal) length. They increase the epithelial surface by a factor of 30 in the small intestine, but are completely absent in the colon. A continuous, postmitotic single layer of intestinal epithelial cells (mostly enterocytes) covers villi and increases the absorptive surface another 600 times due to the presence of microvilli on each cell (Kiela and Ghishan, 2016). Crypts are facing away from the intestinal lumen and sit in invaginations of the intestinal mucosa. They form pockets of approximately 44 μm in diameter and connect to the intestinal lumen only *via* a small opening (around 3 μm), due to dense packing of cells (Maj et al., 2003). The microenvironment within the crypt is further isolated from the digestive process by a continuous outflow of mucus and anti-microbial products. The purpose of this mechanism is to flush potential contaminants out of the crypt and protect the regenerative compartment below. This regenerative zone sits lower in the crypt and consists of a progenitor zone in the crypt middle and a stem cell zone at the very bottom (see Figure 1A). Here, at the crypt bottom stem cells divide unceasingly to fuel the continuous replacement of cells lost at villus tips.

## Simple Yet Diverse – the Intestinal Epithelium

Starting from the crypt bottom toward villus tips we encounter different epithelial cell types in distinct positions. In the following, we will highlight which cells form the intestinal epithelium and how their characteristics and function vary along the crypt villus axis (see Figure 1B).

### Crypt-Base-Columnar Cells

Crypt-base-columnar cells (CBCs) are continuously dividing intestinal stem cells that generate all other epithelial cell types. They reside exclusively at the bottom of crypts wedged between Paneth cells. CBCs divide every 21–24 h in mice and produce two equipotent daughter cells (Dudhwala et al., 2020). Each crypt contains around 15 intestinal stem cells (mouse) although stem cell numbers vary with age. In humans, stem cell numbers are high from birth throughout teenage years, but drop threefold in adults (Dudhwala et al., 2020). In rodents and humans CBCs are identifiable by their expression of LGR5, a receptor for R-spondins (RSPO) (Barker et al., 2007). When LGRs bind RSPOs they prolong and potentiate the action of local WNT signals, which is essential for stem cell maintenance (Hao et al., 2012; Koo et al., 2012).

In a process termed “neutral competition” all stem cells vie for niche space between Paneth cells (Lopez-Garcia et al., 2010; Snippert et al., 2010). Paneth cells, as primary niche cells, provide essential Notch ligands, EGF and (in the mouse) WNT signals. Since Notch signaling can only be induced *via* direct cell–cell contact, membrane contact to a Notch ligand presenting cell in a high-WNT environment is the limiting resource in the stem cell zone. Stem cells that fail to establish niche interactions move up in the crypt to the progenitor zone, where they further differentiate. The continuous competition in the niche is a quality control mechanism that ensures that a healthy stem cell population occupies each crypt. Should a cell suffer DNA damage, a toxic insult or a mutation that reduces its replicative fitness, it will soon be outcompeted by healthy, faster cycling stem cells in the niche and thus expelled from the stem cell zone. Neutral drift dynamics were identified by tracing CBC clones using Confetti – a multicolor labeling strategy. This Cre-loxP based system stochastically recombines a construct containing four inversely arranged fluorescent proteins. Individual clones could then be identified by the expression of a random combination of these fluorescent proteins (Livet et al., 2007). Snippert et al. (2010) used Confetti to follow the fates of clonally labeled LGR5+ cells and demonstrated crypts drifting to monoclonality (become single colored) after an average of 8 weeks. This means that intestinal stem cells appear static in terms of size and positon on a population level but undergo continuous drifts and shifts in clonal composition.

### Paneth Cells

Paneth cells, the primary niche cell for intestinal stem cells, are wedge-shaped secretory cells at the crypt bottom. They contain secretory granules that are filled with antimicrobial products (lysozyme, α- defensins, and phospholipase A2). Low-level release of these products is constitutive, which confers antimicrobial properties to the intestinal mucosa. However, Paneth cells can also drastically increase their secretion in response to IFN-γ, which leads to complete degranulation and extrusion of the cell into the lumen (Farin et al., 2014). The antimicrobial arsenal of Paneth cells gives it broad protective properties against bacteria and even enveloped viruses. Paneth cells do not move with the stream of differentiating cells toward villus tips. Instead, they remain firmly at crypt bottoms due to their expression of Ephrin type-B receptors, which repulses them from the differentiating cell zone, which produces Ephrin-B (Batlle et al., 2002). Paneth cells interact with CBCs on multiple levels. Paneth cell derived EGF and WNT contributes to niche establishment, but is dispensable due to production of the same signaling factors by the surrounding mesenchyme. In fact, WNT production in Paneth cells is limited to the mouse small intestine, since neither the equivalent niche cells in the colon (deep crypt secretory cells) nor human Paneth cells produce the ligand (Sasaki et al., 2016). Notch signals, on the other hand, are (under homeostatic conditions) only provided by Paneth cells at the crypt bottom and are together with availability of WNT stimulation the “limiting factor” that determines stem cell niche size. Despite the fact that Paneth cells provide key signaling molecules to CBCs, their depletion does not result in immediate stem cell loss. CBCs differentiate in absence of Notch signals in high WNT environments directly to Paneth cells (Yin et al., 2014), which causes immediate replenishment of the Paneth cell pool and reestablishment of the niche. Even if diphtheria toxin-mediated Paneth cell ablation is prolonged, alternative niche cells (early enteroendocrine and goblet cells) express DLL1 and can maintain the LGR5^+^ cell pool (Van Es et al., 2019). Earlier studies had indicated that epithelial niche cells might be dispensable altogether, based on intact CBC populations despite complete loss of secretory cells (including Paneth cells) in ATOH^–/–^ animals (Kim et al., 2012). However, loss of ATOH in CBCs artificially simulates continuous NOTCH stimulation (as Notch signaling suppresses ATOH expression), which makes a Notch-ligand expressing niche cell indeed unnecessary. Therefore, these experiments prove redundancy of Paneth cell derived EGF and WNT, but do not conflict with the essential nature of epithelial-niche-derived Notch signals. In fact, the influence of Paneth cells may go beyond direct signaling. A comparison of metabolic activity in CBCs and Paneth cells revealed that the former relied mainly on oxidative phosphorylation, while the latter depended on glycolysis. Lactate, the product of Paneth cell glycolysis, could serve as respiratory substrate for CBCs and contribute to the control of stem cell differentiation *via* ROS induced p38 activation (Rodríguez-Colman et al., 2017).

### Transit Amplifying Cells

Transit amplifying (TA) cells reside in the zone directly above the stem cell zone and are common progenitors of the absorptive lineage. TAs replicate up to six times with an even shorter cell cycle (∼12 h) than CBC cells before they enter a postmitotic state and differentiate further (Potten, 1998). TAs require active Notch signaling in a low-WNT environment to commit to their absorptive fate. Notch ligands are provided by progenitors of the secretory lineage (see below), which induce absorptive fate in all surrounding progenitors in a process termed lateral inhibition. Lateral inhibition maintains a stable ratio between the lineages and ensures that the majority of progenitors will assume absorptive fate and become enterocytes.

### Secretory Progenitors

Similar to TAs, secretory progenitors are direct offspring of CBC cells. They are the common progenitor of the secretory lineage and give rise to Paneth cells, goblet cells, enteroendocrine cells, and Tuft cells. In contrast to TAs, they show a very low proliferative index (Basak et al., 2014). Low mitotic activity is due to lack of Notch signaling in secretory progenitors. HES1, the direct target of Notch activation, is absent and cannot repress the cycle inhibitors *p27KIP1* and *p57KIP2*, which would be essential to maintain a proliferative state (Riccio et al., 2008). Instead, secretory progenitors express ATOH1 (likewise, due to lack of *HES1* repression), which induces expression of Notch-Ligands (DLL1 and DLL4) and thus stimulates Notch signaling (and absorptive fate) in all surrounding cells. ATOH1 is crucial for maintenance of secretory identity, since even specified secretory cells trans-differentiated to absorptive cells when *ATOH1* was depleted (Durand et al., 2012; Kim et al., 2014). The existence of a single multi-potent secretory progenitor population has recently been challenged by the observation that Paneth cells and enteroendocrine cells, but not goblet cells arise from a progenitor population with high non-canonical WNT signaling (Böttcher et al., 2021). Future studies will have to address, whether distinct secretory progenitor populations exist or whether a plastic continuum of secretory progenitors differentiates to individual cell types based on environmental signal inputs.

### Goblet Cells

Goblet cells secrete mucus that lubricates the intestinal lumen and forms a protective layer on the epithelium. Beyond their secretory function, goblet cells can also deliver luminal antigens to dendritic cells to induce tolerance (McDole et al., 2012). Goblet cells are the most numerous among the secretory cells and appear to constitute the default differentiation path for secretory progenitors in absence of other stimuli. In fact, combined inhibition of Notch and WNT signaling is sufficient to completely convert CBC cells to goblet cells (Yin et al., 2014). Recent reports have shed light on the surprising diversity of individual goblet cells. Specialized goblet cells at the crypt opening, so-called sentinel goblet cells, continuously sample the environment by endocytosis. Upon detection of bacterial products (LPS, flagellin, and P3CSK4) these cells release mucus and stimulate other goblet cells lower in the crypt to do the same (Birchenough et al., 2016). Another study identified five distinct goblet-cell types in the human colon in distinct spatial arrangement. Interestingly, the ratios between these goblet-cell populations shifted significantly in patients with ulcerative colitis, indicating a direct link between disease state and goblet-cell fate (Parikh et al., 2019).

### Enteroendocrine Cells

Enteroendocrine (EE) cells are hormone-producing cells that coordinate intestinal functions with the wider organism. Depending on the enteroendocrine cell type and thus the secreted hormonal product, they regulate intestinal motility, satiety, insulin secretion, immune responses, or release of digestive enzymes [for a detailed review, please see Beumer et al. (2020)]. The number of individual EE-cell types is a matter of active debate. Originally, their primary hormonal product classified EE cells. However, improved immunostaining and single-cell techniques revealed multi-hormonal cells and regional differences that do not conform to this definition (Habib et al., 2012; Haber et al., 2017). Recently, real-time-resolved fate mapping identified these multi-hormonal cells as transition stages of trans-differentiation events that occurred during the normal lifecycle of certain EE lineages (Gehart et al., 2019). The authors used Neurog3Chrono, a highly sensitive genetic pulse-chase timer, in conjunction with single-cell sequencing to generate a comprehensive map of enteroendocrine fate with real-time resolution. This map identified key regulators of enteroendocrine differentiation and revealed direct transitions between mature enteroendocrine populations with discrete hormonal profiles as part of normal homeostasis. This enteroendocrine plasticity is closely linked to the movement of EE cells through the changing signaling environment from crypt to villus. BMP signaling, which increases in strength with distance from the crypt bottom, suppressed production of hormones such as GLP1 or TAC1 and promoted expression of villus-enriched hormones such as Secretin or NTS (Beumer et al., 2018). The net result is hormonal zonation, where the same EE cells express and secrete different hormones as they move up the crypt villus axis. However, movement of EE cells appears to be at least partially uncoupled from that of enterocytes. EE cells resided in crypts much longer than absorptive cells. Most EE cells started leaving the crypt at around 60 h after onset of differentiation (Gehart et al., 2019), but individual EE cells remained in the crypt longer than 5 days (Aiken et al., 1994). Next to their primary function, differentiating EE cells could also serve as reserve niche cells for CBCs upon Paneth cell depletion, due to their expression of Notch ligands (Van Es et al., 2019).

### Tuft Cells

With a prevalence around 0.4% of all intestinal epithelial cells, Tuft cells are even rarer than EE cells. Similar to EE cells they are chemosensory cells, but do not produce hormones. Instead, they are closely related to taste receptor cells and express necessary components of taste perception, such as alpha-gustducin and TRPM5. They use their chemosensory ability to initiate type II immune responses in the intestinal epithelium upon detection of parasites, such as helminths or certain protozoa (Gerbe et al., 2016; Howitt et al., 2016; Von Moltke et al., 2016). When a Tuft cell detects parasitic infection (*via* yet unknown ligands) it secretes IL-25 to stimulate IL-13 release in group 2 innate lymphoid cells. This sets off a cascade that imposes strong differentiation bias on intestinal epithelial progenitors and changes epithelial composition to facilitate expulsion of the parasite (for details see “Inflammation-related plasticity” below).

### M Cells

Microfold (M) cells are not evenly distributed along the intestine, but locally concentrated above Peyer’s patches. Peyer’s patches are lymphoid follicles that contain B-, T-, and mononuclear cells and perform immune surveillance. These secondary lymphoid organs are separated from the intestinal lumen by follicle-associated intestinal epithelium (FAE), which differs in cell composition from surrounding intestinal epithelium. FAE is rich in M-cells but lacks goblet cells almost completely. As a result, the mucus layer above the follicle is thinner and allows for better contact with the intestinal lumen. M-cells sample the lumen continuously and transport antigens to the immune cells underneath them. Like all intestinal epithelial cells, M-cells derive from CBC cells. However, they acquire their fate much later than other epithelial cells due to plasticity within the absorptive lineage. All intestinal epithelial cells express the receptor RANK, but RANKL (the ligand) is specifically presented on Peyer’s patches. When absorptive cells encounter RANKL they acquire M-cell fate. Whether only absorptive progenitors or even fully mature enterocytes can switch lineage is not yet clear. However, it is likely that the capacity to trans-differentiate to M-cells is still maintained in mature enterocytes, since exposure to pathogens increased M-cell numbers within few hours (Tahoun et al., 2012). Experiments *in vitro* and *in vivo* have shown that RANKL is both necessary and sufficient to promote M-cell fate (Knoop et al., 2009; de Lau et al., 2012; Kanaya et al., 2012). However, M-cell differentiation could be blocked by nociceptor sensory neurons *via* release of CGRP. Although the exact mechanism of the M-cell reduction upon neuronal activation is not clear yet, its purpose is to limit the number of M-cells as entry points upon Salmonella Typhimurium infection (Lai et al., 2020).

### Enterocytes

Enterocytes are the most common cells of the intestinal epithelium. Their primary role is the controlled transport of nutrients, water and ions from the intestinal lumen into the body. Until recently, enterocytes along the crypt villus axis were considered homogeneous. However, single-cell studies discovered several types of enterocytes with distinct functions at specific positions along the crypt-villus axis. With the help of laser capture microdissection (LCM) Moor et al. (2018) created a large panel of landmark genes that was subsequently used to align an intestinal epithelial single cell dataset along the villus. Thus, the team uncovered spatial zonation of absorptive cells and concluded that each enterocyte moved up the crypt-villus axis and trans-differentiated into several enterocyte types in the course of its limited lifetime of 3–4 days. At the bottom of the villus, enterocytes express an anti-microbial program and specialize in amino-acid transport. Mid-villus enterocytes are the main transporters for carbohydrates and upper-villus enterocytes are responsible for lipid uptake. The signals that underlie the formation of these functional gradients are not clearly identified yet, but similar to EE cells a direct link to BMP signaling is likely. First insights come from identification of specific villus-tip telocytes that provide BMP and non-canonical WNT signals to tip-enterocytes. Ablation of these telocytes resulted in loss of some, but not all, tip-enterocyte markers in the tip-epithelium (Halpern et al., 2020).

**FIGURE 2:**
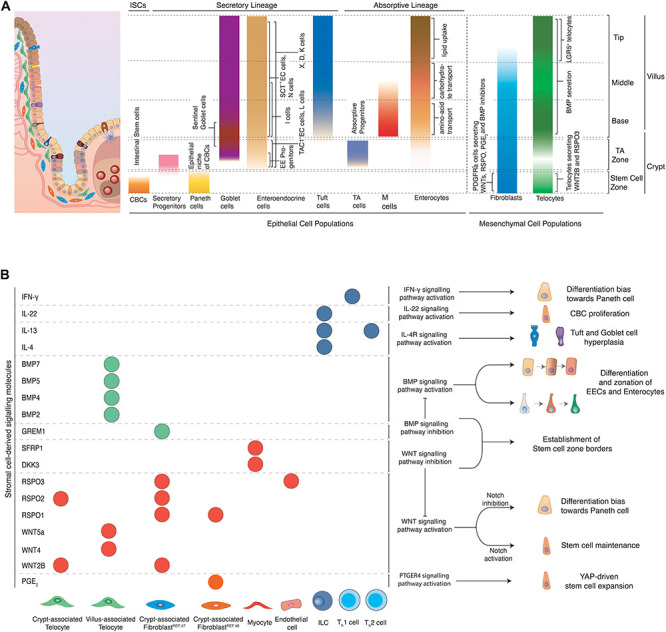
**(A)** Topology of epithelial and mesenchymal cell populations across the crypt-villus axis. Distinct populations of epithelial and mesenchymal cells can be encountered at specific positions along the crypt villus axis. CBCs located at the crypt bottom, proliferate and can give rise to all epithelial cell types of the intestine. Secretory populations exist at various positions across the crypt-villus axis, including Paneth cells (crypt bottom) that protect and nurture CBCs, Tuft (villus) and goblet (crypt + villus) cells that coordinate inflammatory responses, as well as hormone-producing enteroendocrine cells (crypt + villus). Absorptive progenitors give rise to enterocytes and M cells. Enterocytes located at different parts of the villus are linked to distinct functions such as amino-acid (aa) and carbohydrate transport and lipid uptake. M cells are mainly located above Peyer’s patches and their main role is to transport antigens to the antigen-presenting cells underneath them for further processing. Stromal cells provide structural support to the tissue and provide epithelial cells with signaling molecules, regulating important processes such as proliferation and differentiation. Several fibroblast populations located at the crypt bottom in close proximity to the stem cell zone have been linked to production of WNTs and RSPO, which are essential for stem cell maintenance. Telocytes have varying secretory profiles depending on their position along the crypt-villus axis. A subset of telocytes found under the crypt produce canonical WNT ligands and RSPO3. However, telocytes locally concentrated at the villus base and tips and are linked to production of BMP ligands that promote differentiation of epithelial cells. **(B)** Effects of stromal cell-derived signals on intestinal epithelial cells. Stromal cells produce various signaling molecules affecting the behavior of intestinal epithelial cells. Telocytes and fibroblasts located near the stem cell zone secrete WNT ligands and RSPO to maintain stemness of CBCs, while WNT antagonists and BMP inhibitors, produced by myocytes and crypt-associated telocytes establish the limits that distinguish the stem cell zone from the rest of the crypt. Upon damage, fibroblast-derived PGE_2_ drives the regeneration of stem cells *via* the YAP signaling axis. BMPs produced mainly by telocytes found in the villus induce differentiation and zonation of enterocytes, enteroendocrine cells and potentially other cell types as they migrate from the villus base toward the tip. Likewise, inflammatory signals derived from immune cells drive stem cell expansion and proliferation, instruct cell fate decisions and introduce strong differentiation biases toward secretory cell lineages so that tissue’s homeostasis is re-established after damage of the intestinal epithelium.

In summary, most intestinal epithelial cell types show distinct spatial patterns of occurrence and function along the crypt-villus axis (see Figure 2). Similar observations have been made in other organs, such as the liver, where hepatocyte functions change significantly from the central vein region to the portal triad zone. However, whereas the liver is almost static in cellular composition during normal homeostasis, the intestine shows fast, directional-flow turnover. This necessitates a high degree of plasticity, where individual cells contribute to different zones and thus purposes in the course of their lifetime. To ensure robust zonation, the instructions to undergo these fate changes have to be provided either by the epithelium itself (e.g., lateral inhibition) or by a well-structured stromal environment. The full extent of intestinal micro-structure and plasticity-induced zonation has yet to be comprehensively revealed. Interestingly, recent years have seen the development of a number of new technologies that would be well suited to study these spatio-temporal relations in the intestine.

### Looking Beyond the Field – Upcoming Technologies to Investigate Spatial Relations in the Intestine

The anatomy of the gastrointestinal tract and the composition of its different regions have traditionally been studied with techniques that provided limited information of its actual micro-structure or dynamics. Recently developed spatio-temporal techniques have overcome these limitations by increasing the available spatial and temporal resolution. However, they are yet to be applied to the study of the gastro-intestinal tract. In the following paragraph we look beyond the field of intestinal biology and identify techniques and applications that could be useful to deepen our understanding of the gut.

**FIGURE 3:**
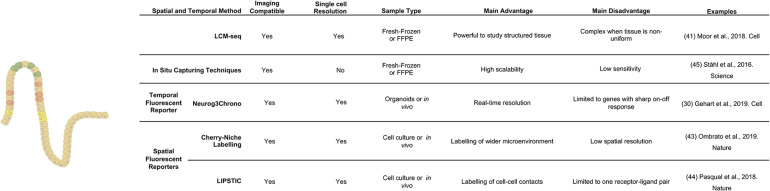
Comparison of the main methods to study spatio-temporal relationships.

Newly developed spatial and temporal reporter systems have the ability to highlight cell interaction and to follow cell fate in time. So far, only the Neurog3Chrono system found application in the intestine (see enteroendocrine cells). Recently, a promising tool for niche identification has been developed in the field of cancer biology. The sLP-mCherry niche labeling system was used to label environments during breast cancer metastasis in the lung (Ombrato et al., 2019). The authors engineered cells to release a cell-penetrating mCherry fluorescent protein that labeled nearby cells *in vivo*. A similar strategy could be employed for studying intestinal stem cell niches or the specific environments that induce fate transitions along the crypt-villus axis. The Victora group took a different approach to discern direct cell interactions: they fused bacterial sortase A to a receptor on the surface of one cell population of interest and five N-terminal glycines to the corresponding ligand on the surface of another cell population of interest. When these two populations encountered each other in presence of a fluorescent or biotin marked substrate the fluorescent (or biotin) mark was transferred in an enzymatic reaction to the ligand-presenting cell. This method called LIPSTIC has been used to study the dynamic interactions of T-cells and dendritic cells, but could also be utilized to resolve specific cell interactions (e.g., alternative stem cell niche cells) in the intestinal epithelium *in vivo* (see Figure 3; Pasqual et al., 2018).

Parallel to reporter systems, modified scRNA-seq techniques with inherent spatial resolution have gained traction. Spatial transcriptomics is an RNA-seq based approach that analyses transcriptomes at thousands of individual spots across a histologic tissue section (Ståhl et al., 2016). Due to its unbiased nature, this approach has the potential to become a powerful tool to study tissue microstructure. Currently the method is still held back by limited spatial resolution (around three cells) and strongly reduced sensitivity in comparison to standard single cell RNA sequencing. In the gastrointestinal field spatial transcriptomic has recently been applied in combination with scRNA-seq to explore the early development of the human intestine (Fawkner-Corbett et al., 2021), but similar studies in adult tissue that characterize intestinal microstructure and its changes in various disease states are still missing.

Overall, there is a wide variety of upcoming methods to interrogate the temporal and spatial dimensions of the gastrointestinal tract. Nevertheless, generation of a comprehensive picture of the dynamic changes in intestinal function during health and disease will remain a challenge. Future improvements to the scalability of multimodal methods (techniques that simultaneously measure multiple parameters in a single cell) or methods that aim at integrating data from different tools will be crucial to paint a comprehensive picture of position, fate and function. Particularly mesenchymal structure is an important part of the equation. Due to its role in instructing stem cell maintenance and differentiation, the mesenchyme is an equally structured component of the intestine that has yet to receive more attention.

## More Than Stroma – the Intestinal Mesenchyme

The intestinal stroma consists of several cell types, namely fibroblasts, myofibroblasts, pericytes, telocytes, endothelial cells, neural cells, and immune cells. Altogether, they provide structural support to the tissue and produce signals that are essential for stem cell maintenance, self-renewal and tissue zonation (see Figure 1). Mesenchymal cells produce diverse signaling molecules depending to their position across the crypt-villus axis. Stromal cells near the crypt bottom, where CBCs reside, mainly produce WNT ligands, RSPOs, and BMP inhibitors which block differentiation and support maintenance of stemness. Mesenchymal cells that are located above the crypt produce a rising BMP gradient toward the villus that induces maturation of CBC daughter cells and accounts for zonation of EE cells and most likely enterocytes (see Figure 2).

Mesenchymal cell populations in close proximity to the crypt have been investigated extensively for their potential role in constituting the niche of CBCs. A subset of PDGFRa^+^; CD34^+^ fibroblasts located near the crypt base produce canonical WNT2B, RSPO1, RSPO2, and RSPO3 (McCarthy et al., 2020). This population serves also as the main source of the BMP antagonist GREM1. As a result, these cells enhance WNT but inhibit BMP signals in the crypt. Thus, they provide two essential components for stem cell maintenance (see Figure 2). The niche function is further corroborated by the ability of the same cell population to support the formation and passaging of organoids in co-culture experiments in the absence of RSPO (a crucial WNT signaling potentiator) or NOGGIN (an otherwise necessary BMP inhibitor) (McCarthy et al., 2020). In addition, a rare (potentially overlapping), PDGFRa^+^ and CD34^+^ population of fibroblasts located around the crypts, in close proximity to the stem cell zone has been recently linked to production of Prostaglandin E_2_ (PGE_2_) (see Figure 2). Fibroblast-derived PGE_2_ binds to its receptor expressed in intestinal stem cells and induces the activation of a YAP transcriptional program, which drives the expansion of stem cells. In co-culture experiments, this population induced increased stem cell identity in organoids (Roulis et al., 2020). Other mesenchymal cells positive for PDGFRa and CD34 lie above the crypt, but do not produce BMP inhibitors, which underscores the exquisite microstructure in the mesenchyme that enables epithelial zonation. Independently, pericryptal myofibroblasts marked by PDGFRa expression produce WNT ligands and RSPO3. In fact, presence of these cells in co-culture makes addition of RSPO to small intestinal organoids obsolete. When *Rspo3* is specifically ablated from these myofibroblasts in co-culture, organoid growth is significantly reduced (Greicius et al., 2018). It is apparent that many stromal populations can support organoid formation, however, whether the populations described are identical or partially overlapping remains to be further elucidated. In the colon, a sub-epithelial mesenchymal population expressing GLI1 provides WNT signals for CBCs. When *Wntless*, which encodes a protein required for the secretion of WNT ligands, is genetically ablated in these stromal cells, LGR5^+^ CBCs are lost and the integrity of the colonic epithelium is impaired. GLI1-expressing cells are also present in the small intestine, with a major role as a reserve source of WNT, when it is not sufficiently provided to stem cells by epithelial cells (Degirmenci et al., 2018). The importance of mesenchymal WNT is likely more pronounced in humans than mice, since mouse Paneth cells do produce sufficient WNT to maintain stem cells, while their human equivalents do not. This is evidenced in organoid culture, where addition of WNT to the medium was only necessary for human small intestinal organoids.

Another mesenchymal cell type of sub-epithelial cells, termed “telocytes,” has recently received increased attention due to its complex role in epithelial patterning. Telocytes are large cells with extended processes called “telopodes,” embedded in the basal lamina between the capillary plexus and the intestinal epithelium and are characterized by the expression of FOXL1 (Shoshkes-Carmel et al., 2018). Although telocytes can be found along the whole crypt-villus axis, their density is higher in the villus base and tips (see Figure 2). Bulk sequencing detected production of canonical WNT2B, non-canonical WNT4, WNT5A, WNT5B, RSPO3, and several BMP ligands such as BMP2, BMP3, BMP4, BMP5, and BMP7 in these cells. However, expression of both BMPs and WNT ligands in the same population was paradoxical, since one pathway promoted differentiation, whereas the other induced stemness. Subsequent spatial analysis of telocytes *via* single-molecule Fluorescence *in situ* Hybridization (smFISH) identified several distinct telocyte populations with strikingly different expression profiles. Telocytes located near crypts produced canonical WNT2B and RSPO3, while non-canonical WNT5A and BMP5 were enriched in telocytes found in the crypt-villus junctions. More importantly, blocking WNT secretion from telocytes by genetic inactivation of *Porcn*, resulted in reduced proliferation of CBCs in both small and large intestine and reduced WNT signaling. This indicated that telocytes are a critical source of WNTs for epithelial cell proliferation (Shoshkes-Carmel et al., 2018; McCarthy et al., 2020). Moreover, an LGR5^+^ subpopulation of telocytes located at villus tips has been recently described (see Figure 2). These LGR5^+^ telocytes produced BMPs and WNT5a, which suggested that non-canonical WNT signaling may play a role in establishing tip identity. Genetic ablation of these tip-telocytes led to loss of most tip-specific enterocyte markers. These markers only returned after 3 weeks, when also LGR5^+^ telocytes had reappeared. This identified the LGR5^+^ telocyte population as major regulator of the late enterocyte transcriptional program (Halpern et al., 2020).

However, the intestinal epithelium is not shaped by fibroblast and telocyte-derived products alone. Intestinal endothelial cells have been recently linked to production of RSPO3 to maintain intestinal homeostasis (see Figure 2B; Ogasawara et al., 2018). On the other hand, myocytes produce WNT antagonists, namely DKK3 and SFRP1 that may limit WNT signaling activation above the stem cell zone and thus induce differentiation of stem cell progeny (McCarthy et al., 2020). Moreover, distinct immune cells are linked to secretion of inflammatory cytokines that drive CBC proliferation and differentiation to secretory cells in order to maintain tissue integrity and homeostasis (see Figure 2B and “inflammation-related plasticity below”).

A major limitation of our current knowledge of mesenchymal populations and their spatial organization is the poor comparability of results. Without a more comprehensive approach it is difficult to ascertain, whether individual studies describe the same or differing cell populations. Additionally complexity comes from regionalization along the gastro-intestinal tract. It is very likely that the well-described regional differences from proximal to distal small intestinal epithelium are equally reflected in different mesenchymal populations. A coordinated effort with standardized methods, such as unbiased, spatially resolved single cell sequencing, will be needed to unlock the full complexity of the stromal structure that informs intestinal identity. Beyond identification and mapping of mesenchymal populations, functional assays are crucial to determine effects of epithelial-mesenchymal interactions. Thankfully, faithful *in vitro* tissue modeling has become more accessible in the last decade due to the development of organoid technology.

## *In vivo* Systems To Assess Niche Function and Epithelium-Mesenchyme Interactions

**FIGURE 4:**
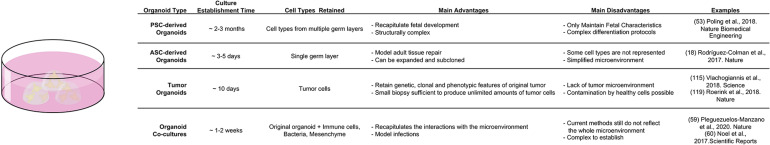
Comparison of different organoid systems to assess fate determination and plasticity in homeostasis and disease.

*In vivo* studies of epithelial-mesenchymal interactions are inherently difficult, due to low accessibility and high complexity of native tissues in a living organism. This is why *in vitro* techniques, such as organoid technology see increased use in mechanistic exploration of basic tissue function. “Mini guts” give researchers the opportunity to simulate intestinal function, regeneration and disease in a dish as organoids recapitulate the cell type-composition, general structure and self-renewal process of their tissue of origin. They can be obtained either from pluripotent stem cells (either embryonic or induced), or multipotent adult stem cells (LGR5+ CBC cells). Either has distinct advantages, when exploring epithelial-mesenchymal interactions. Organoids derived from Pluripotent Stem Cells (PSC) recapitulate fetal development of the intestine and are excellent tools to study this process *ex vivo*. In addition, the guided differentiation of PSCs fosters co-development of epithelial and mesenchymal tissue, which provides important insights into co-dependencies of both layers. For example, PSC-derived human intestinal organoids have been used to study how mechanical forces that are necessary for intestinal development induce transcriptional changes that are crucial of correct maturation of epithelium and mesenchyme (Poling et al., 2018). However, the differentiation procedure from PSCs to intestinal tissue is complex. It usually takes an average of 2–3 months for the organoids to develop fully and, unless transplanted under the kidney capsule, they maintain fetal characteristics (McCracken et al., 2011) [for an in depth look at PSC derived endodermal organoids please refer to Kechele and Wells (2019)]. In contrast, adult stem cell derived organoids model adult tissue repair and solely consist of epithelium. This lack of mesenchymal structures reduces the system complexity, but also enables the investigation of deliberate, artificial environmental changes thanks to the defined nature of organoid media. This makes adult intestinal organoids a powerful system to investigate individual signaling molecules that can be simply added or withdrawn from the defined medium. Likewise, adult “mini-guts” can be employed to study epithelial-mesenchymal interactions in well-defined co-culture assays. Multiple studies that identified mesenchymal niche cell populations have used adult intestinal organoid co-culture to demonstrate niche-function of mesenchymal populations (Stzepourginski et al., 2017). A similar approach in the stomach used gastric organoid co-culture and single cell-sequencing to identify a particular LGR5+ fibroblast population as main source of RSPO3 in the tissue (Chen et al., 2019). Recently, the complexity of co-culture systems has been expanded even further, as both immune cells and bacteria have been added to mini-intestines, which greatly expands the possibilities for future uses of the system (Figure 4; Noel et al., 2017; Bar-Ephraim et al., 2020; Pleguezuelos-Manzano et al., 2020).

Beyond studying environmental interactions, organoids also find applications in exploring the inherent self-organization of tissues. Serra et al. (2019) used adult intestinal organoids to describe how single cells generate multicellular asymmetric structures and discovered a critical role of YAP-1 in the process. Furthermore, adult organoids were employed to describe how the metabolic activity of LGR5+ CBCs and Paneth cells play a role in supporting optimal stem cell function in the intestine (Rodríguez-Colman et al., 2017). In general, the ease of establishment (3–7 days), accessibility and expandability (split rates of 1:3 to 1:4 each week) make adult epithelial organoids excellent tools to study the effects of specific manipulations of an otherwise fully defined system. Yet, the simplicity of the system is also its limitation. Even current co-culture systems only add a single cell type at a time, which prevents cross-talk between mesenchymal or immune populations. Future efforts will have to develop more advanced co-culture systems that bridge the gap between impenetrable complexity *in vivo* and over-simplified interactions *in vitro*.

## Cellular and Tissue Plasticity in the Intestinal Epithelium

At its core, plasticity describes the ability of individual cells or whole tissues to change their function dynamically in response to extrinsic factors. On a tissue level, extrinsic factors like diet, inflammatory signals or tissue damage change the cellular composition of the intestinal epithelium. These factors either directly affect differentiation decisions on stem cell level or induce cellular plasticity in mature populations. Both de-differentiation and trans-differentiation fall under the umbrella term of cellular plasticity. The former describes a process during which mature cells return to a progenitor/stem cell state, whereas the latter implies direct conversion from one mature cell type to another (Tetteh et al., 2015). In any tissue, the factors promoting and limiting plasticity need to be well balanced to confer adaptability and robustness at the same time. If this balance tips toward stability, the tissue may be unable to regenerate after injury, if it tips toward plasticity, cancer may ensue.

The intestinal epithelium is a highly plastic epithelium that can rapidly respond to metabolic, inflammatory or regenerative challenges. The adaptability of the intestine serves on the one hand to balance function with energy expenditure and on the other hand to ensure epithelial integrity. It comes in the form of trans-differentiation, as intestinal cell populations change their function in response to environmental stimuli and their position along the crypt-villus axis, but also in the form of de-differentiation, when regenerative capacities are exhausted. The intestine is lined by 30 m^2^ of single-layered epithelium that shields the rest of the organism from 10^13^ bacteria in its lumen. Due to its thinness, the barrier is ideal for nutrient uptake, but lacks the strength to withstand mechanical abrasion and environmental insults repeatedly. This is why continuous self-renewal, though energy expensive, is necessary to maintain epithelial integrity. Self-renewal depends on the presence of continuously dividing, healthy stem cell populations that provide a steady flow of replacement cells. However, unceasing cell division makes stem cells also susceptible to DNA damage, radiation and cytotoxic substances. Consequently, a variety of mechanisms (seclusion in crypts, neutral competition, and spatial niche limitations) is in place to protect stem cells and prevent malignant transformation. Likewise, an extensive backup system, in the form of intestinal plasticity, enables the intestine to re-establish homeostasis rapidly after catastrophic stem cell loss. Thus, both trans- and de-differentiation are integral components of normal intestinal function.

### Metabolic Plasticity

**FIGURE 5:**
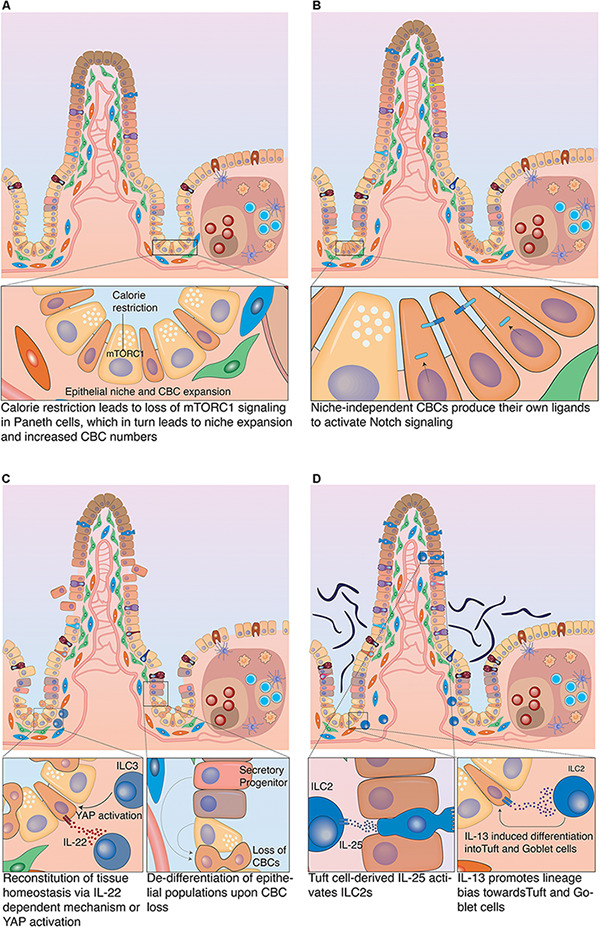
Plasticity of the intestinal epithelium upon different challenges. **(A)** Calorie restriction. Long-term fasting induces morphological changes in the intestine associated with reduced villus-length. It also affects the stem cell zone, by inducing an increase in the populations of CBCs and Paneth cells and decrease in TA cells. **(B)** Nutrient overabundance. High-fat diet affects the stem cell compartment, as it induces an increase in the number of CBCs and decrease in Paneth cells. This was linked to the acquisition of Notch independence by CBCs as they produce their own Notch ligands to stimulate Notch signaling. **(C)** Damage-induced plasticity. Severe damage of the epithelium can lead to profound inflammation that in turn activates group 3 innate lymphoid cells (ILC3), which produce IL-25 to support CBC proliferation. Alternatively, ILC3s can also promote tissue regeneration by CBCs *via* an IL-25 independent mechanism, which involves the activation of YAP signaling in epithelial cells. This effect is most likely mediated by a stromal population that reacts to ILC3 activation with release of IL-11. If CBCs have been damaged or eliminated in the course of the insult, differentiated epithelial cells can fall back into the niche and de-differentiate to restart tissue replenishment. **(D)** Infection-related plasticity. Upon infection, Tuft and goblet cells are activated to produce anti-microbial products. Also, Tuft cells secrete IL-25 that activates ILC2s which in turn secrete Il-13. IL-13 acts on epithelial cells and strongly favors differentiation to Tuft and goblet cells, which results in Tuft and goblet cell hyperplasia.

The intestinal epithelium has a fast cell turnover that requires significant energy expenditure to maintain. Consequently, such energy-expensive process has to be well balanced with actual caloric intake, particularly if an organism undergoes prolonged periods of fasting. During starvation, the snake’s intestine undergoes atrophy, a condition associated with reduced intestinal mass, as intestinal surface area and epithelial cell numbers are significantly reduced. Upon re-feeding, rapid and extensive remodeling occurs when the intestinal turnover is restarted (Secor et al., 1994). Analogous mechanisms have also been described in mammals: long-term fasting caused atrophy in the rat intestine leading to a reduction in villi length, which was reversed upon re-feeding (Dunel-Erb et al., 2001). This shortening of villi was also reflected in changes in the regenerative compartment. Food withdrawal caused an increase in the number of Paneth cells and thus CBCs (due to increased niche space). Furthermore, it induced a decrease in TA cells, concomitant with overall reduced proliferation. Interestingly, calorie restriction was associated with reduction of mTORC1 signaling in Paneth cells (see Figure 5A). Whether the detected loss of mTORC1 signaling in Paneth cells was directly responsible for the increase in their numbers, remains to be clarified. However, this mechanism was strongly suggested by the fact that forced activation of mTORC1 in Paneth cells prevented niche and stem cell expansion upon starvation. This identified the niche as main detector of metabolic status and regulator of stem cell numbers upon limited nutrient availability (Yilmaz et al., 2012). Whereas the reduction in proliferation conserves energy, the increase in CBC numbers may poise the tissue for immediate regeneration, once nutrients are available. Additional regenerative capacity upon re-feeding rests in reserve stem cell populations (often referred to as +4 cells). Nutrient deprivation induced PTEN inhibition in reserve stem cells (mostly progenitors of the secretory lineage) and an increase in their number (Richmond et al., 2015). Surprisingly, mice on high-fat diet also showed elevated numbers of CBCs. In contrast to fasting, however, the number of Paneth cells was decreased. This finding was counter-intuitive, since stem cells depend on Notch signals that are only provided in direct membrane contact with Paneth cells during homeostasis. However, this contradiction was explained by the fact that high fat diet induced expression of Notch ligands in CBCs, which allowed them to act as their own primary niche cells and uncoupled them from Paneth cells (see Figure 5B). Interestingly, this created a direct link between high caloric intake and carcinogenesis, since niche independence is the first important step that ensures survival of malignant cells. The nutritional status exerted its effect on stem cells *via* PPARδ signaling (Beyaz et al., 2016). Consistent with this assumption, pharmacological activation of PPARδ mimicked the high-fat response and granted non-ISC populations the capacity to form tumors upon APC loss (Beyaz et al., 2016). Recently, another link between cell fate determination and metabolism has been described. Loss of *Lkb1* in LGR5^+^ cells induced a differentiation bias toward the secretory lineage and thus boosted the number of secretory cells (Gao et al., 2020). During homeostasis LKB1 inhibits PDK4, which would otherwise block pyruvate dehydrogenase. Pyruvate dehydrogenase is a key enzyme in oxidative phosphorylation on which CBCs rely metabolically. When *Lkb1* was ablated oxidative phosphorylation was decreased, which resulted in upregulation of *Atoh1* mRNA levels, which in turn promoted an increase in the number of secretory cells (Gao et al., 2020). Likewise, loss of the pyruvate carrier *Mpc1* in LGR5^+^ cells resulted in increased proliferation and expansion of the stem cell compartment (Schell et al., 2017). This expansion was likely caused by increased fatty acid metabolism, which translated to stabilization of β-catenin and increased WNT signaling (Beyaz et al., 2016). Although these genetic loss-of-function models induced artificial metabolic changes, they clearly show that the metabolic state of CBCs can dynamically control proliferation as well as cell fate decisions. Future studies will have to address to which extent, circadian metabolic fluctuations and diet composition directly affect stem cell function.

### Inflammation-Related Plasticity

Similarly to nutrition, inflammation-mediated signals play a significant role in regulating intestinal cell plasticity and have strong impact on CBC behavior. Inflammatory responses can be initiated by tissue damage or infection. In both cases, specialized immune cells are activated and secrete factors that support re-establishment of tissue homeostasis. Group 3 innate lymphoid cells (ILC3) are present in the intestine in close proximity to crypts. ILC3s react to tissue injury and secrete IL-22, which has been implicated in epithelial regeneration of the intestine. IL-22 activated the JAK-STAT signaling pathway in CBCs, which supported stem cell survival and proliferation in response to damage (Lindemans et al., 2015). Additional, IL-22 independent mechanisms have been identified that support crypt cell proliferation during intestinal tissue regeneration *via* the Hippo pathway (Romera-Hernández et al., 2020). ILC3s activated YAP signaling in LGR5^+^ CBCs to support the regeneration process of the tissue. YAP signaling plays key roles in the regenerating intestine, as loss of the pathway results in a defective regeneration process (Barry et al., 2013; Gregorieff et al., 2015). ILC3s were necessary for the activation of YAP signaling, since no YAP response occurred in crypts of mice lacking ILC3s after Methotrexate (MTX)-induced intestinal damage. Mechanistically, YAP activation can be induced *via* the IL-6 family receptor GP130. The GP130 receptor can dimerize with IL-6 or IL-11 receptors (IL-6R and IL-11R) to form functional receptor dimers that respond to their respective ligands (Taniguchi et al., 2015). Although LGR5^+^ cells express GP130 and IL-11RA1, ILC3s do not directly produce IL-11. Instead, IL-11 is known to be produced by other stromal cells. This implies the involvement of another stromal population as an intermediate between epithelial cells and ILC3 activation (see Figure 5C; Romera-Hernández et al., 2020).

Upon infection with parasites, such as helminths, the intestinal epithelium presents with granulomatous infiltrates containing different immune cells, including natural killer cells (NK cells), neutrophils and innate lymphoid cells. However, not only immune cells but also mucus-producing goblet cells and chemo-sensory Tuft cells are heavily involved in the intestinal response to parasitic infection. In addition to producing anti-microbial molecules, Tuft cells respond to helminth infections by producing IL-25, which in turn activates tissue-resident group 2 innate lymphoid cells (ILC2). ILC2 cells produce IL-13 that binds to its receptor IL-4Rα expressed in LGR5^+^ stem cells and DLL1^+^ secretory progenitors. IL-13 signaling in these cells induces strong lineage bias in the differentiation process that favors production of Tuft and goblet cells. This leads to a profound goblet and Tuft cell hyperplasia, which is crucial to facilitate the successful expulsion of the nematode from the intestine (see Figure 5D; Gerbe et al., 2016; Von Moltke et al., 2016). Interestingly, helminth infection had a strong impact on the transcriptional profile of stem cells beyond the aforementioned lineage bias. Crypts in direct proximity to granulomatous infiltrates lost stem cell marker expression such as LGR5 and OLFM4, despite continuing proliferation. CBCs in these crypts displayed an IFNγ signaling signature, which was associated with expression of *Sca-1* and fetal markers such as *Gja1* and *Spp1*. Culturing these SCA-1^+^ epithelial cells as organoids, led to the formation of spheroids that lacked markers of differentiated epithelial cells (Nusse et al., 2018). Furthermore, these spheroids were insensitive to RSPO withdrawal from medium, which had also been demonstrated in cultures of mouse fetal intestinal epithelium (Mustata et al., 2013). This indicates that CBCs can return to a partially fetal state under influence of a changed microenvironment during parasitic infection. To which extent this fetal reversion benefits re-establishment of tissue integrity and function is not yet fully established (Nusse et al., 2018).

Another study has recently shed light on the crosstalk between LGR5^+^ cells and T-helper cells (T_*h*_ cell) (Biton et al., 2018). Two subpopulations of LGR5^+^ cells were identified *via* scRNA-seq analysis, that express MHC class II proteins (MHC II) and can activate T cells as antigen-presenting cells. The authors showed in organoid culture experiments that multiple inflammatory signals affect ISC proliferation and differentiation in contrasting ways. More specifically, co-culture of intestinal organoids with T regulatory cells (T_*regs*_) or IL-10, their secretory product, induced the expansion of CBCs. In contrast, co-culture with T_*h*_1 or supplementation with exogenous IFN-γ resulted in the differentiation of CBCs to Paneth cells. Conversely, T_*h*_2 co-cultures or addition of IL-13 promoted the differentiation of CBCs to Tuft cells. Moreover, deletion of MHCII in LGR5^+^ cells prevented remodeling of the tissue upon infection with pathogenic *H*eligmosomoides *polygyrus* and induced an increase in LGR5^+^ cell numbers, which disrupted the mucosal immune response (Biton et al., 2018). Overall, these data suggest that CBCs play a role in regulating the tissue’s adaptive immunity by responding to pro-inflammatory and anti-inflammatory signals with Paneth or Tuft cell differentiation respectively. This highlights the crosstalk of immune cells with stem cells as a mechanism to re-establish and maintain tissue homeostasis upon different inflammatory conditions.

### Cellular Plasticity During Regeneration of the Intestine

Despite the “proliferative vulnerability” of its stem cells, the intestinal epithelium possesses a remarkable ability to recover from severe stress such as irradiation, or chemotherapy. In fact, the resistance of the intestinal epithelium surpasses that of tissues with quiescent stem cells such as the bone marrow (Withers and Elkind, 2009). Even when stem cells are completely lost in the course of the insult, CBCs re-appear and are the main contributors to regeneration after injury. This is possible, since differentiated cells re-acquire CBC cell status when in contact with an empty niche space. This process relies equally on the instructive capacity of a dynamic stem cell niche and plasticity in the epithelium (see Figure 5C).

Due to the role of LGR5^+^ CBCs as stem cells during intestinal homeostasis and their importance in regeneration, several studies have tested their necessity for intestinal regeneration by depleting them *via* irradiation or Diphtheria toxin (DT)-mediated ablation (Tian et al., 2011; van Es et al., 2012). Interestingly, in all cases LGR5^+^ cells re-appeared within 2–3 days after complete removal. However, when their resurgence was blocked due to continuous DT-mediated ablation, the regeneration process failed (Metcalfe et al., 2014; Tan et al., 2021). This implies that the LGR5^+^ cell pool is essential for intestinal regeneration, but can be replenished by an alternative cell source. This replenishment could come either from a dedicated reserve stem cell population or from de-differentiation of more mature populations (see Figure 1B). This question has recently been addressed in an elegant study that investigated intestinal recovery from irradiation with short-term lineage tracing. By limiting the timeframe of lineage labeling, the authors ensured that only recently generated cells would inherit a fluorescent mark and potential long-lived, quiescent reserve stem cell populations would not. Subsequent ablation of CBCs revealed that all re-appearing stem cells carried the fluorescent label. This clearly indicated that LGR5^+^ cells were replenished from their recent progeny undergoing de-differentiation, rather than a reserve stem cell population. Interestingly, both absorptive and secretory lineage cells could contribute to the recovery of the CBCs (Murata et al., 2020). The lack of evidence for a dedicated reserve stem cell population has shifted the research focus more and more toward plasticity. Indeed, the replenishment of LGR5^+^ cells has been attributed to various alternative sources, ranging from secretory progenitors and enterocyte progenitors, to more differentiated cell types such as EECs. One of the first studies describing that lineage-committed cells could revert to stem cells, when LGR5^+^ cells were depleted, identified DLL1^+^ secretory progenitors as source of new CBC cells (van Es et al., 2012). Due to their low proliferation index, secretory progenitors are likely to withstand insults that mainly affect dividing cells such as CBCs and TA cells. This low division rate also explains why former approaches to identify quiescent stem cells primarily identified cells with secretory characteristics. For example, genetic labeling of long-lived intestinal cells with low turnover with an elegant split-Cre-system revealed a reserve stem cell population that gave rise to the secretory lineage under homeostasis but could revert to CBC cells upon damage (Buczacki et al., 2013). It is more than likely that both Dll1-lineage tracing and label retention experiments revealed the same cell population of secretory progenitors. Thus, the concept of plasticity reconciles reports of a quiescent +4 stem cell populations with the CBC stem cell model. However, plasticity is not limited to secretory progenitors. Multiple studies attributed the ability to acquire stem-cell like features to Paneth cells when CBCs were lost (Yu et al., 2018; Jones et al., 2019). Genetic labeling of Paneth cells and subsequent irradiation-induced stem cell depletion revealed lineage tracing of Paneth cells, suggesting that they are able to de-differentiate. This was further supported by their ability to form organoids and by analysis of their transcriptional status, which revealed stem cell-like expression profiles (Yu et al., 2018; Jones et al., 2019).

Likewise, Tuft cells marked by DCLK1 expression can contribute to recovery of intestinal injury. Upon loss of APC, Tuft cells can also initiate the formation of adenocarcinomas in a DSS-colitis model (Westphalen et al., 2014). Additional de-differentiation capability has been attributed to EECs. *Bmi1* and *Prox1* based tracing of the early EEC lineage by Yan et al. (2017) showed extensive conversion to stem cell fate upon tissue damage. The ability of lineage-committed cell populations to de-differentiate is not limited to secretory cells, as it extends even to the upper crypt, where enterocyte progenitors are located. TA cells, which generate mature enterocytes, are marked by the expression of alkaline phosphatase (ALPI) and were also capable of de-differentiation upon targeted ablation of LGR5^+^ stem cells (Tetteh et al., 2016).

Although de-differentiation of multiple committed cell types in the intestine has been demonstrated, the exact mechanism and order of events during the de-differentiation process is unclear. Profiling of the epigenetic status of LGR5^+^ cells and their progeny, revealed that there were no significant differences between them at the level of DNA methylation and histones (Kim et al., 2014; Jadhav et al., 2017). This lack of epigenetic changes during differentiation certainly facilitates the observed plasticity in the intestinal epithelium. However, we still lack mechanistic insight into the de-differentiation process and the instructive role of specific niche components. ASCL2 has been recently identified as requirement for successful recovery of the intestine after lethal damage to CBCs. In fact, ASCL2 expression was found to be specifically induced in intestinal epithelial cells, before they fell back into the stem cell zone and acquired LGR5 expression. Single-cell RNA-seq revealed that ASCL2^+^ cells lacked expression of *Clusterin*, a marker of the recently described population of revival stem cells that were activated when the intestine was damaged by irradiation (Ayyaz et al., 2019), but expressed markers of EE and goblet cells. This suggested that these cells represented a transition state between mature cell and stem cell. Molecular analysis revealed IL-11RA as a direct target of ASCL2 and its upregulation in ASCL2^+^ regenerating crypt cells. Indeed, supplementation of IL-11 in organoid cultures of sorted ASCL2^+^ cells enhanced their spheroid formation ability, which suggests that ASCL2^+^ cells depended on the IL-11 signaling axis for proliferation in order to facilitate the regeneration of the damaged intestine (Murata et al., 2020). Further studies will have to investigate the full extent of signals and pathways that induce these de-differentiation events. To this end, further characterization of the stem cell microenvironment during a de-differentiation stimulus would be of particular importance, to define essential regulators of the process and delineate how controlled plasticity could be utilized for regenerative medicine. Likewise, new lineage-tracing technologies will be necessary to study the quantitative contributions to de-differentiation of each cell type and to establish where the limits of plasticity lie. Interestingly, several new approaches have been recently developed that greatly increase the capabilities of classic lineage tracing experiments.

### Looking Beyond the Field – Upcoming Methods for Studying Lineage, Differentiation, and Plasticity

Plasticity, differentiation, and particularly de-differentiation events have been predominantly studied *in vivo* with classic Cre-lox based lineage tracing. In these experiments a fluorophore or lacZ is activated in a population of interest and the same label is inherited by all offspring. Although several improvement to the system have been made [e.g., Brainbow/Confetti system to distinguish up to 100 individual clones (Livet et al., 2007; Cai et al., 2013)] the general experimental setup and readouts (primarily imaging) have remained the same. Recently, the increased accessibility of sequencing technology and genome editing have created powerful alternatives to the classic lineage tracing experiment. In the following paragraph we look beyond the field of gastrointestinal biology and identify upcoming technologies that could deepen our understanding of lineage and plasticity in the gut.

**FIGURE 6:**
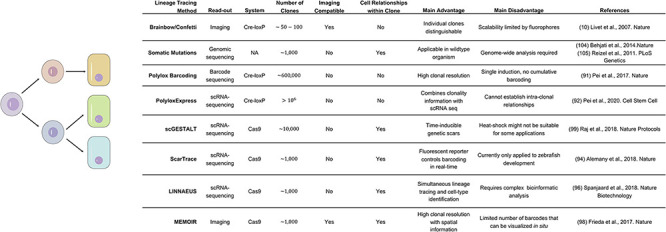
Comparison of the main technologies available to perform lineage tracing.

DNA or RNA barcoding strategies can easily overcome the limited number of labels that can be distinguished in fluorescence-based clonal identification. Whereas the first techniques to adopt barcoding still relied on Cre recombinases [e.g., Polylox and PolyloxExpress (Pei et al., 2017; Pei et al., 2020)] the field is predominantly switching to Cas9-based barcoding. The main reason for the switch lies in the difference of modification kinetics, with the Cre recombinase acting too fast to allow for progressively evolving labels that can later be used to reconstruct the order of events. CRISPR-Cas9 techniques, on the other hand, can utilize differing affinities of individual sites to tune modification speed and thus prolong the timeframe of lineage recording. Directed to a specific genomic locus by a guide RNA Cas9 nuclease generates a double strand break (DSB), which can lead to small insertions or deletions (indels). The continuous increase in indels across 10 or 100 s of potential targeting sites can then be used to establish the clonal history of each cell after the genomic regions containing the barcodes have been sequenced (McKenna et al., 2016; Alemany et al., 2018; Kalhor et al., 2018; Spanjaard et al., 2018). However, since a gRNA will no longer bind its target site once it is mutated, the number of scars that can be induced and thus the timeframe of recording and the complexity of the clonal information is inherently limited. To overcome this limitation, Church and colleagues developed mSCRIBE. By engineering a guide RNA that targeted its own spacer sequence, it was possible to perform multiple rounds of scarring over a longer period (Kalhor et al., 2017). Furthermore, alternative strategies have been developed to combine CRISPR-Cas9 scarring with additional readouts. On the one hand, MEMOIR used multiple transgenes that could be visualized with seqFISH (a high-throughput smFISH technique), adding a spatial dimension to the technique (Frieda et al., 2017). On the other hand, several techniques integrated a CRISPR-Cas9 strategy with RNA-seq. This brings the great advantage that cell state and clonal history can be established in a single step (Alemany et al., 2018; Raj et al., 2018). However, all CRISPR-Cas9 based lineage tracing techniques share the same limitation. The generation of the barcoding indels causes DSBs, which are toxic to many cells and could bias the result of an experiment toward more resistant cell populations (see Figure 6; Baron and van Oudenaarden, 2019; Wagner and Klein, 2020). Despite this limitation, barcoded lineage tracing could find wide application in intestinal biology. Clonal dynamics during neutral competition could be explored in thousands of clones in parallel. Combination with single cell sequencing could detect the existence of lineage bias in particular stem cell clones and the molecular mechanisms behind it. Finally, de-differentiation could be studied to quantify the individual contributions of each cell type in the process. However, particularly the last application needs an additional step in technology, since cell state and not only barcodes will need to be written into DNA at the beginning of the tracing. Interestingly, CRISPR has great potential not only as a gene editing tool, but also as a molecular recorder. Recently, Platt and his team made use of the system’s capacity to acquire RNA and integrate it into a CRISPR array in a sequential manner. This approach makes it possible to sample a cell’s RNA pool upon activation and store the information genetically (Tanna et al., 2020). Current applications of the method are still limited to bacteria and the obtained information is very sparse, but this promising technology could pave (upon further development) the way to internal recording of the transcriptional states of mammalian cells (Schmidt et al., 2018).

All so far mentioned tools necessitate genetic modification of the traced organism. Therefore, they are suitable to study model systems but not humans. In order to study lineage tracing without genetic interventions, the continuous accumulation of somatic mutations in each cell can be utilized. Either single nucleotide variants (SNPs) or microsatellite mutations, which are mostly functionally neutral, can be followed (Behjati et al., 2014). For example, microsatellites have previously been used to recapitulate the clonal evolution during the development of colonic crypts (Reizel et al., 2011). However, both SNPs and microsatellites are rare and scattered across the genome. Therefore, genome-wide sequencing approaches are necessary, which are expensive and still difficult to apply on single cell level (see Figure 6). Overall, in the gastro-intestinal system these methods may prove useful to validate the vast body of knowledge on clonal dynamics and plasticity that has been primarily generated in the mouse. Although several studies followed fixation and expansion of individual mutations in human colon (Nicholson et al., 2018; Baker et al., 2019), a comprehensive study of these dynamics in human subjects is still missing. Changes in clonal dynamics are particularly important in the field of intestinal cancer, where mutated cells first outcompete healthy stem cells in their own crypt before expanding laterally by crypt-fission, long before additional mutations will cause an overt malignancy. Next generation lineage-tracing approaches have the ability to follow the expansion of thousands of mutated subclones in parallel. This will enable researchers to determine the true extent of clonal variance in the intestinal epithelium and to study the nature of the competitive advantage of individual clonal populations that will eventually cause cancer.

### Plasticity and Cancer: Two Sides of the Same Coin

In a healthy crypt cells compete for limited niche signals that are required for maintenance of stemness. The size of the niche controls the number of stem cells and the point of differentiation onset. It does so, *via* gradients of signaling molecules that either promote stemness (e.g., WNT and EGF) or differentiation (e.g., BMP). For cancer to occur, epithelial cells need to develop independence from niche-derived proliferative signals and resistance to differentiation stimuli. Vogelstein and colleagues have proposed a model describing the adenoma-carcinoma sequence, as well as defining the genetic alterations that contribute to colorectal cancer progression. As one of the first steps, constitutive activation of WNT signaling (e.g., by loss of APC), is thought to be necessary for tumor initiation. Progression depends on activating mutations in EGFR pathway components, such as KRAS, and inactivating mutations in p53 and the TGF-β/BMP signaling pathway component SMAD4 (Fearon and Vogelstein, 1990; Muzny et al., 2012). It is thus apparent that colorectal cancer depends on the abnormal activation of signaling pathways that control stem cell identity and maintenance. In fact, stepwise genome editing in colon organoids demonstrated that three mutations in the main signaling pathways of the intestinal niche (WNT, EGF, and BMP signaling) together with loss of p53 were sufficient to transform a healthy epithelial cell to an invasive cancer cell (Drost et al., 2015). Whereas healthy stem cells are limited by the spatial restrictions of the niche, cells that acquire the aforementioned mutations achieve niche-independence. In contrast to other cancers, colon cancer has a relatively narrow set of common mutations. In part, this may be due to continuous competition for niche space. Since mutations are acquired sequentially, a mutated sub-clone needs to constantly outcompete healthy stem cells in the course of its repeated mutagenesis. This means, that only mutations that provide an increased proliferative fitness [such as KRAS (Muzny et al., 2012)] are permissive. Any mutation that reduces proliferative fitness, e.g., by prolonging the metaphase or reducing productive cell division will be quickly lost from the crypt. Once an epithelial cell has acquired niche independence, its offspring can outgrow normal tissue limits and form a tumor. However, even in a tumor, cells differ in proliferative capacity and differentiation status (de S. e Melo et al., 2017; Lenos et al., 2018). From these differences arose the concept of Cancer Stem Cells (CSCs). Indeed, lineage tracing studies revealed that LGR5^+^ cells were capable of tumor initiation and gave rise to all tumor cells (Schepers et al., 2012). However, genetic ablation of LGR5^+^ cells in mouse intestinal tumor organoids (tumoroids) by the administration of DT restricted primary tumor growth but did not result in tumor regression. Moreover, once DT was withdrawn, LGR5^+^ cells reappeared immediately. Similarly, LGR5^+^ cell depletion in human intestinal tumoroids, by insertion of an inducible Caspase-9 construct into the Lgr5 locus caused regression. However, when the chemical agent that induced caspase activation was no longer administered, LGR5^+^ cells re-appeared. The authors elucidated that differentiated tumor cells characterized by expression of KRT20, could revert to LGR5^+^ cells, to fuel tumor growth (Shimokawa et al., 2017). This suggested, that similarly to the normal tissue, tumor growth was driven by cells in a stem cell state. However, the same plasticity that enabled healthy tissue to recover from catastrophic stem cell loss, also enabled more differentiated tumor cells to re-acquire stem cell characteristics (de S. e Melo et al., 2017). Recent studies have shed more light onto components of the normal tissue stem cell niche that enable plasticity (Murata et al., 2020). The mechanisms that enable plasticity in a tumor are far less understood. Lenos et al. (2018) have shown that the CSC phenotype was adopted by cells located at the tumor edge, near cancer associated fibroblasts (CAFs). Although CSC markers were expressed throughout the tumor, only CSCs at the tumor edges displayed clonogenicity. However, re-transplantation of CSCs obtained from the center of xenografted tumors indicated that these cells could also effectively drive tumor growth, suggesting that functionality of CSCs was regulated by microenvironmentally derived signals and that tumor cell position was of particular importance for clonal expansion. CAFs produce Osteopontin (OPN), which enhanced *in vivo* proliferation of CSCs located in the outer part of the tumor, where OPN concentrations were higher. Overexpression of OPN in tumor cells that were transplanted, accelerated tumor growth compared to respective controls and was sufficient to drive clonogenic growth of tumors independently of CAFs (Lenos et al., 2018). Besides tumor growth, CAF-derived signals have also been implicated in cancer initiation. A fibroblast subpopulation located near the crypts, in close proximity to the stem cell zone, produces PGE_2_. PGE_2_ binds to its receptor PTGER4, expressed in stem cells of the crypt, which leads to de-phosphorylation of YAP and activation of YAP target genes (Roulis et al., 2020). Active YAP signaling drives the expansion of a stem cell population characterized by the expression of SCA-1 also termed reserve stem cells (Roulis et al., 2020). This signaling network was shown to be involved in tumor formation, as genetic ablation of *Ptgs2*, which catalyzes the conversion of Arachidonic Acid to Prostaglandins, in fibroblasts or genetic ablation of *Ptger4* in intestinal epithelial cells led to the formation of significantly fewer tumors in a mouse model of colorectal cancer. Interestingly, the growth of already established tumors was not affected, as tumor volumes did not differ from the respective controls, which suggested that this fibroblast-derived tumorigenic signal was necessary for tumor initiation but not for tumor growth (Roulis et al., 2020). These studies provided evidence that the microenvironment has a crucial role in regulating stem cell states during normal homeostasis and carcinogenesis. Tumor initiation does not rely solely on cell-intrinsic properties (e.g., mutations), but also requires a finely orchestrated environment. Interactions within this tumor microenvironment remain poorly elucidated. This creates a need for elegant tools that enable their comprehensive characterization on single cell level. Understanding the plasticity promoting mechanisms at the interface between tumor and normal tissue may open new therapeutic avenues to prevent cancer progression.

In this context, organoids are a promising *in vitro* system that enables researchers to study and compare normal tissue regeneration and cancer development. Several studies have shown that cancer organoids (or tumoroids) share the same clonal heterogeneity, the same resistances and the same vulnerabilities as their tumor of origin (Vlachogiannis et al., 2018; Boretto et al., 2019; Yao et al., 2020). Additionally, organoids can model the same plasticity that is observed in tumors. de S. e Melo et al. (2017) used mouse intestinal cancer-derived organoids as a model to demonstrate how tumor cells compensate for the loss of CSCs by de-differentiation of non-CSC populations. In addition, Fumagalli et al. (2020) used LGR5-reporter cancer organoids to prove that the majority of metastases are formed by LGR5- (non-CSC) tumor cells that acquire LGR5+ (CSC) identity upon engraftment at the metastatic site. The switch from non-CSC to CSC state was indeed necessary for efficient metastatic outgrowth. Beyond the mechanistic exploration of cancer biology, organoid tumor models can also be used in the context of personalized cancer medicine. In fact, several studies have shown that the organoid response *in vitro* is predictive for the patient response (Vlachogiannis et al., 2018; Boretto et al., 2019; Yao et al., 2020). Thanks to the expandability of tumoroid cultures, even a small biopsy generates sufficient tumoroid tissue for functional assays like drug screening. Alternatively, the ability to grow individual tumor subclones can be utilized to study tumor heterogeneity on a functional level. When Roerink et al. (2018) established around 60 clonal tumoroid lines from three colon carcinomas they found functional differences in drug responses that would not have been predictable, based on epigenetic, genomic, and transcriptomic data alone. This study emphasized the need for functional experiments to tailor treatment to individual patients. However, the application of organoids for personalized cancer medicine still faces significant challenges. Although, tumoroids can be expanded in culture, the time from biopsy to assay remains in a range of 2–3 months due to the required amount of tissue (see Figure 4). Therefore, significant technological improvements will be necessary to make tumoroid based personalized medicine compatible with the necessary swiftness of therapeutic decisions. In the future, *in vitro* drug screening assay may also need to account for plasticity in tumor cells, since drug susceptibilities inherently change when cells transition from CSC to non-CSC states. An increased understanding of the environments that induce these changes in cell identity and how to model them *in vitro* could therefore further improve our ability to correctly predict disease outcome in intestinal cancer.

## Discussion

The growing number of techniques to study cellular relations in space and time have already transformed our understanding of tissue function in health and disease. Combined lineage-tracing, single-cell and organoid experiments have revealed surprising plasticity in the intestine. During health and disease, intestinal epithelial cells undergo de- and trans-differentiation that is integral to tissue function. The process creates zonation, allows for metabolic adaptation and spatially separates intestinal processes. Likewise, it gives the intestine surprising resistance against toxic, inflammatory, or irradiation insults. Cellular plasticity and particularly de-differentiation is not limited to the intestine. A growing number of reports finds de-differentiation events in a wide range of epithelial tissues (Freedman et al., 2013; Pan et al., 2013; Stange et al., 2013; Tata et al., 2013; Raven et al., 2017). This suggest that cellular plasticity plays a much larger role in adult mammalian organisms than currently appreciated. Technological improvements in single-cell methodology and upcoming lineage tracing methods will be crucial for gaging the true extent of functional and regenerative flexibility in mature tissues.

Plasticity complicates traditional models of stemness, maturity, and cell types. Already now, the classic, hierarchical differentiation tree of discrete, binary decisions seems incompatible with biological reality. Instead, a dynamic model emerges, where cells re-evaluate their identity continuously as a function of extrinsic pushes toward and intrinsic resistance against fate change. Resistance to fate change is a product of past environmental inputs that resulted in long-lasting cellular changes (e.g., epigenetic modification). With increasing epigenetic distance between two cell states the transition resistance grows, but can still be overcome by a strong enough trans-differentiation or de-differentiation signal. In the intestine the extent of epigenetic changes in the course of differentiation is surprisingly small, which certainly contributes to the high levels of intestinal plasticity (Kim et al., 2014; Jadhav et al., 2017). This low fate-change resistance is coupled with highly instructive signaling zones along the crypt-villus axis. One of the most potent ones, the stem cell niche, can overcome the de-differentiation resistance of differentiated progenitors (van Es et al., 2012; Tetteh et al., 2016) and most likely even mature cells (Westphalen et al., 2014; Yan et al., 2017; Yu et al., 2018; Jones et al., 2019). Continuous, overlapping signaling gradients that stretch along the crypt-villus axis give each height increment a unique signaling environment. These environments instruct changes in the cells that move through them, which generates the diversity of the intestinal epithelium. Cell identity is thus not discrete but a wide spectrum of states with differing function. Enterocytes, enteroendocrine cells, goblet cells and most likely other cell types traverse through these identity spectra in the course of their lives. This allows the intestinal epithelium to retain static functional zonation despite the continuous movement of the epithelial cell sheet.

Although plasticity in the intestine has been convincingly demonstrated several important questions remain to be answered: Which epithelial and mesenchymal signals and cell populations shape the diversity along the crypt-villus axis? Which environmental signals induce and limit de-differentiation of mature cells? Do de-differentiated cells retain features of their former state and does this memory cause lineage bias? How can plasticity be utilized to enhance tissue regeneration? And how can plasticity in cancer be prevented to limit therapy escape? Spatially and temporally resolved reporter systems, spatial transcriptomics and advanced Cas9-based lineage tracing tools will be crucial in answering these questions. However, the vast amount of data that these tools can produce have to be validated and translated into mechanistic understanding. This is why, the transition from descriptive to functional exploration of niche environments is equally important. In this regard, organoids are a very powerful tool that is ideally suited to complement single-cell-resolved *in vivo* experiments. Their capacity to replicate the microarchitecture, functionality, and cellular diversity makes them ideal to study tissue self-organization and microenvironments. Mapping the dynamic changes in these microenvironments will enable us to understand the general and tissue specific principles of regeneration and tumor progression. Thus, we may be able to replenish the regenerative capacity of stem cells or prevent malignant cells from escaping the limits of homeostasis, not by directly targeting them, but by reshaping their environments.

## Author Contributions

VB, CR, and HG researched and wrote the manuscript. HG reviewed and edited the manuscript before submission. All authors contributed to the article and approved the submitted version.

## Conflict of Interest

The authors declare that the research was conducted in the absence of any commercial or financial relationships that could be construed as a potential conflict of interest.
